# Surgical approach, relapse risk and overall survival in borderline ovarian tumors: age-stratified analysis based on real-world data

**DOI:** 10.3389/fonc.2026.1810193

**Published:** 2026-04-07

**Authors:** Karina Liuba, Natalia Maciuszko, Sara Alson, Mihaela Asp

**Affiliations:** 1 Department of Obstetrics and Gynecology, Skåne University Hospital, Lund, Sweden; 2 Department of Clinical Science, Lund University, Lund, Sweden; 3 Department of Obstetrics and Gynecology, Ystad Hospital, Ystad, Sweden

**Keywords:** borderline ovarian tumor, complete staging, conservative surgery, Cox regression, recurrence, survival

## Abstract

**Background:**

The need for complete staging surgery including hysterectomy for all age patients with borderline ovarian tumors (BOT) is debatable, considering fertility in younger patients and potential higher morbidity in older patients. We aimed to investigate the relapse risk and overall survival in a cohort including patients who underwent conservative surgery (organ-preserving procedures) to minimize complications, independent of fertility considerations.

**Methods:**

We conducted a retrospective, population-based review of 217 surgically treated BOT patients in Southern Sweden (2017–2022). Patients were stratified by age (≤40, 41–50, 51–65, >65 years) and surgical approach (conservative vs complete staging). Outcomes included recurrence, 5-year overall survival (OS), progression-free survival (PFS) and OS at end-of-follow-up. Kaplan–Meier analyses with log-rank tests evaluated survival differences; univariate and multivariate Cox proportional hazards models identified independent predictors.

**Results:**

Of 217 patients, 9 had a synchronous malignancy and were excluded from the survival analyses. 36 underwent conservative surgery, 167 complete staging surgery and 5 bilateral salpingo-oophorectomy with uterus preservation. Recurrence was more frequent after conservative surgery (12.1%) than complete surgery (3.2%) but did not reach statistical significance. Five-year OS was 100% for patients ≤40 and 41–50 years, 95.3% for those 51–65 years and 92.5% for >65 years (log-rank p=0.02). PFS differed by age in the overall cohort (p=0.002) and among complete surgery patients (p=0.001), but not in conservative surgery patients (p=0.721). OS did not differ significantly among complete surgery patients across age categories (p=0.287). In multivariate Cox regression, age at first operation was the only independent predictor of survival (HR = 1.068, 95% CI 1.018–1.119, p=0.007).

**Conclusion:**

Age at first operation independently predicts survival in BOT. Young age at first surgery was associated with a higher risk of relapse, regardless the type of surgery, whereas older age was associated with an increased risk of death. Conservative surgery does not compromise long-term outcomes and can be considered across age categories to balance oncologic safety with the goal of reducing surgical morbidity, supporting individualized treatment planning.

## Introduction

Borderline ovarian tumors (BOT) account for approximately 10% to 20% of all epithelial ovarian cancers (1, 2). As the term *borderline* implies, they exhibit clinical behavior that is intermediate between benign and malignant tumors. Histologically, this is characterized by cellular proliferation and nuclear atypia without destructive stromal invasion (3, 4).

BOT have a favorable prognosis but current management presents challenges due to variable recurrence risk and patient heterogeneity across age groups (5). Complete staging surgery, often including hysterectomy, is traditionally recommended to minimize recurrence; however, this approach may increase perioperative and long-term morbidity, particularly in older patients.

Due to the early stage at diagnosis and the younger age of affected patients, fertility-sparing surgery is the preferred approach. This method, which preserves ovarian tissue and the uterus, is considered a safe option for younger women despite a slightly higher risk of recurrence (6). Additionally, preserving ovarian tissue in women under the age of 50 is important even when fertility is not a concern, as early menopause can have a negative impact on women’s health (6, 7).

Historically, organ-preserving procedures have been termed ‘fertility-sparing surgery’ and offered primarily to younger women. In the present study, we deliberately use the term ‘conservative surgery’ to encompass organ preservation performed for broader clinical reasons, including reduction of surgical complications in elderly patients for whom fertility is not relevant. Evidence regarding the oncologic safety of conservative surgery across age categories remains limited.

We aimed to compare recurrence and survival outcomes between conservative and complete staging surgery across four age strata (≤40, 41–50, 51–65, >65 years), and to identify independent predictors of survival using multivariate Cox regression.

To address clinically meaningful differences in fertility potential, endocrine function, and age-related health status, we stratified women into four age categories: ≤40, 41–50, 51–65, and >65 years. Existing literature primarily distinguishes between fertility-preserving and non–fertility-preserving surgery, leaving an important gap regarding women who no longer desire pregnancy but remain premenopausal, where ovarian preservation continues to have physiological relevance. Our age categories were therefore chosen to reflect biological and clinical transitions rather than previously established groupings.

Women aged ≤40 years typically retain reproductive potential and may still have a desire for pregnancy, making fertility considerations central to surgical decision-making. The 41–50-year group represents women who are generally beyond reproductive intentions but remain premenopausal, where preservation of ovarian endocrine function may influence long-term health and quality of life. The 51–65-year category corresponds to early postmenopausal age, when ovarian function has ceased but overall health status remains relatively stable. Women >65 years were separated as a distinct group due to the increasing likelihood of comorbidities, frailty and declining performance status, factors that may influence both surgical risk and postoperative outcomes.

This stratification thus captures clinically relevant physiological stages across the menopausal transition and later life, enabling a more nuanced assessment of outcomes than fertility-based categories alone.

We hypothesized that conservative surgery would not compromise overall survival while potentially reducing procedure-related morbidity in older patients.

## Materials and methods

### Study design and setting

This retrospective, population-based study was conducted in the Southern Region of Sweden after approval from the Swedish Ethical Review Authority (approval number: 2023-03384-01). The study period covered surgeries performed between January 1, 2017 and December 31, 2022.

### Participants and data sources

Eligible patients underwent surgery for suspected BOT. Those not operated at the tertiary oncologic gynecology center (Department of Obstetrics and Gynecology, Skåne University Hospital, Lund) were referred to the Lund Pathology Department for second histopathological review and diagnostic confirmation. Cases were identified via the Laboratory Information Management System (LIMS) by querying BOT diagnoses. Clinical data were extracted from the electronic medical record (Melior; Cerner Corporation, Kansas City, USA).

### Variables and definitions

Collected variables included age at first operation, obstetric history, body mass index (BMI), CA 125 levels, FIGO stage (when applicable), primary diagnosis at recurrence, date of operation, surgical approach (conservative surgery, restaging or radical/complete staging), recurrence status and date, and survival status. Histopathological diagnoses were based on formalin-fixed, paraffin-embedded specimens reviewed by specialized gynecologic pathologists. In this manuscript ‘conservative surgery’ denotes organ-preserving procedures (uterus and at least one ovary retained) irrespective of fertility intentions; ‘complete staging’ denotes removal per standard staging protocols (including hysterectomy when indicated). In several patients, the diagnosis was unexpected at the time of the initial procedure. When these patients subsequently underwent restaging surgery, classification followed the definitive procedure: if restaging surgery was radical (bilateral oophorectomy, with or without hysterectomy), the patient was assigned to the radical surgery group. If an ovary or part of an ovary was preserved, with or without hysterectomy, the patient was assigned to the conservative surgery group.

### Outcomes

Primary outcomes were overall survival (OS) and progression-free survival (PFS). Secondary outcomes included recurrence rate and 5-year OS. Survival analyses were stratified by age categories: ≤40, 41–50, 51–65, and >65 years.

Relapse-free survival (RFS) (alternatively progression-free survival, if applicable) was defined as the time from the date of primary surgery to the date of first documented recurrence. Patients without recurrence at the end of follow-up were censored at the date of last clinical assessment. Overall survival was used, as cause-specific mortality data were not consistently available in this retrospective cohort. Overall survival (OS) was defined as the time from the date of primary surgery to the date of death from any cause. Patients who were alive at the end of follow-up were censored at the date of last contact. Missing data were limited; patients with substantial missing information were excluded, whereas isolated missing variables were coded and the patients retained in the analyses.

### Statistical analysis

Analyses were performed using SPSS version 20.0 (IBM Corp., Armonk, NY, USA). Continuous variables were compared using Student’s t-test or nonparametric tests, according to distribution. Kaplan–Meier curves were generated for PFS and OS, and group differences assessed by log-rank tests. Cox regression analyses were performed as both univariate and multivariate models, adjusting for ECOG performance status, type of surgery, histological subtype, and age. Statistical significance was set at p<0.05.

### Ethics

The study was approved by the Swedish Ethical Review Authority (approval number: 2023-03384-01). The research was conducted in accordance with the Declaration of Helsinki and relevant national regulations.

## Results

### Patient population and clinical characteristics

Between 2017–2022 a total of 263 patients were referred to the Pathology Department at Lund University Hospital, a tertiary center for gynecological cancers. Most patients were initially operated on in the southern region of Sweden, but for those who were not operated on at Lund University Hospital, histological samples were sent for a second opinion. All patients had a clinical suspicion of BOT or borderline-related ovarian cancer. Of these, 170 patients (64.6%) underwent surgery at Lund University Hospital, while the remaining 93 patients (35.4%) underwent surgery at regional hospitals.

Among the 263 referred patients, 217 (82.5%) had a confirmed diagnosis of BOT at the tertiary center. Nine patients with borderline tumors and synchronous malignancies were excluded from survival analyses. The clinicopathological characteristics of the total cohort and the subgroup with confirmed BOT are summarized in **Table 1**.

**Table 1:** Clinical characteristics of the ovarian borderline tumors population.

Variables	Total	Serous borderline	Mucinous borderline	Endometroid borderline	*p*	Borderline in ovarium plus synchronous malignancy*
Number patients (%)	217	132 (60.8%)	73 (33.8%)	3 (1.4%)	**0.00**	9 (4.1%)
Age median (range)	54 (17-90)	55 (17-90)	52 (17-86)	54 (33-78)	0.68	59(50-69)
BMI median (range)	26 (16-58)	26 (26-50)	26 (16-58)	24 (20-42)	0.67	31 (22-36)
Ca125 median (range)	73 (10-3275)	137 (10-3275)	53 (13-384)	44 (12-76)	**0.04**	658.5 (17-1300)
HE4 median (range)	77.5 (1-1100)	69 (34-334)	75 (1-319)	214 (77-352)	0.35	601 (102-1100)
Endometriosis	27 (12.44)	21(18.9)	5 (8.1%)	1 (33.33%)	0.14	0
ECOG 0	183 (92.42%)	113 (91.9%)	62 (92.5%)	3 (100%)	0.96	4 (100%
Missing data	18					
Children before first surgery					0.43	
0	58	38 (28.3%)	20 (28.2%)	2 (66%)		0
1	78	22 (17.3%)	56	0		0
≥2	71	69		1 (33%)		9 (100%)
Missing data	10					
Pregnancy after first surgery					**0.00**	0
1	11	9 (6.6%)	2 (2.7%)	0		
2	3	1 (0.7%)	1(1.4%)	1 (25%)		
Children after first surgery					**0.00**	
1	8	7(5.5%)	1 (1.4%)			
≥2	2	1(0.7%)	1 (1.4%)	1 (33.3%)		0
Relapse free	205 (94.5%)	124 (93.9%)	70 (95.9%)	3 (100%)	0.78	3 (88.9%)
Five years survival (alive)	207 (94.5%)	125 (94.7%)	71 (97.3%)	3 (100%)	0.62	3 (88.9%)
Alive at the end of the study	203 (93.5%)	124 (93.9%)	70 (95.9%)	3 (100%)	0.09	6 (66.6%)

BMI, Body Mass Index; CA 125, Cancer Antigen 125; HE4, Human Epididymis Protein 4; SOEB, Salpingo-oophorectomy; ECOG, Eastern Cooperative Oncology Group Performance Status.

Bold value significate statistical significance (<0.05).

*Cases with a synchronous malignancy were excluded from the analyses.

The median age of patients with confirmed BOT was 54 years (range 17–90), with no significant differences among histological subtypes (serous 55 years, mucinous 52 years, endometrioid 54 years; p=0.68). Median BMI was 26 (range 16–58) across subtypes (p=0.67). Preoperative CA-125 levels were higher in serous tumors (median 137 U/mL) compared with mucinous (53 U/mL) and endometrioid tumors (44 U/mL), reaching statistical significance (p=0.04), while HE4 levels did not differ significantly between subtypes (p=0.35).

Endometriosis was present in 27 patients (12.4%), most frequently in serous tumors (18.9%), but this difference was not statistically significant (p=0.14). The majority of patients had good performance status (ECOG 0, 92.4%), with no significant differences across histological subtypes (p=0.96).

Conservative surgery was performed in 36 patients (18%), most commonly in patients with endometrioid tumors (66.7%), although differences across subtypes were not significant (p=0.14). Among patients, 58 had no children, 78 had one child, and 71 had two or more children prior to surgery. Postoperative pregnancies occurred in 14 patients (38,8%) with 10 subsequent live births (27,7%).

Relapse-free survival was high overall (94.5%), with no significant differences among histological subtypes (p=0.78). Similarly, overall five-year survival was 94.5%, and 93.5% of patients were alive at the end of the study, with no significant differences across subtypes (p=0.09).

### Impact of surgical intent on patient survival

A total of 208 patients were included in the survival analysis of surgical intent, comprising 36 patients who underwent conservative surgery, 167 patients who underwent complete staging surgery and 5 patients who underwent bilateral salpingo-oophorectomy (SOEB) as the primary procedure (**Table 2**). The median age differed significantly across surgical groups: 35 years (range 17–90) for conservative surgery, 61 years (range 20–87) for complete staging, and 28 years (range 25–87) for SOEB (p=0.001).

**Table 2:** Impact of surgical intent on patient survival.

Type of surgery (patients)	Total N=208	Conservative surgery N = 36 (17.3%)	Complete staging N=167 (80.4%)	p	SOEB* N=5 (2.4%)
Age-years (median/range)	54 (17-90)	35 (17-90)	61 (20-87)	0.001	28 (25-87)
Relapse (patients)					
Yes	11(5.3%)	4 (11.4%)	6 (3.6%)	0.094	1 (20%)
No	296 (94.7%)	31 (88.6%)	165 (96.4%)		4 (80%)
Missing	1	1			
5 Years Survival#	96	18	78	0.43	
Yes	92 (95.8%)	18 (100%)	74 (94.4%)		4 (80%)
No	4 (4.2%)	0	4 (5.1%)		1 (20%)
Alive at the end of the study				0.59	
Yes	197(94.7%)	34(94.4%)	159(95.2%)		4 (80%)
No	11(5.3%)	2(5.6)	8(4.8%)		1 (20%)

*SOEB, Salpingo-oophorectomy.

#Some patients did not reach five-year follow-up due to the study period ending in 2022.

Relapse occurred in 11 patients (5.3%) overall, with 4 patients (11.4%) in the conservative surgery group, 6 patients (3.6%) in the complete staging group, and 1 patient (20%) in the SOEB group. The differences in relapse rates among surgical groups were not statistically significant (p=0.94).

Of the 208 patients included in the study, only 96 were eligible for five-year follow-up because the study period ended in 2022. The analysis of five-year survival is therefore based on this subgroup.

Among the 96 patients with available five-year data, 92 (95.8%) were alive at five years, while 4 (4.2%) had died before the five-year mark. When comparing conservative vs complete surgery groups (n=18 and n=78), the five-year survival was 100% (18/18) in the first group and 94.4% (74/78) in the second. In the SOEB subgroup (n=5), the five-year survival was 80% (4/5), with one patient (20%) deceased. There was no statistically significant difference between the groups (p=0.43, **Table 2**).

**Figure 1:**
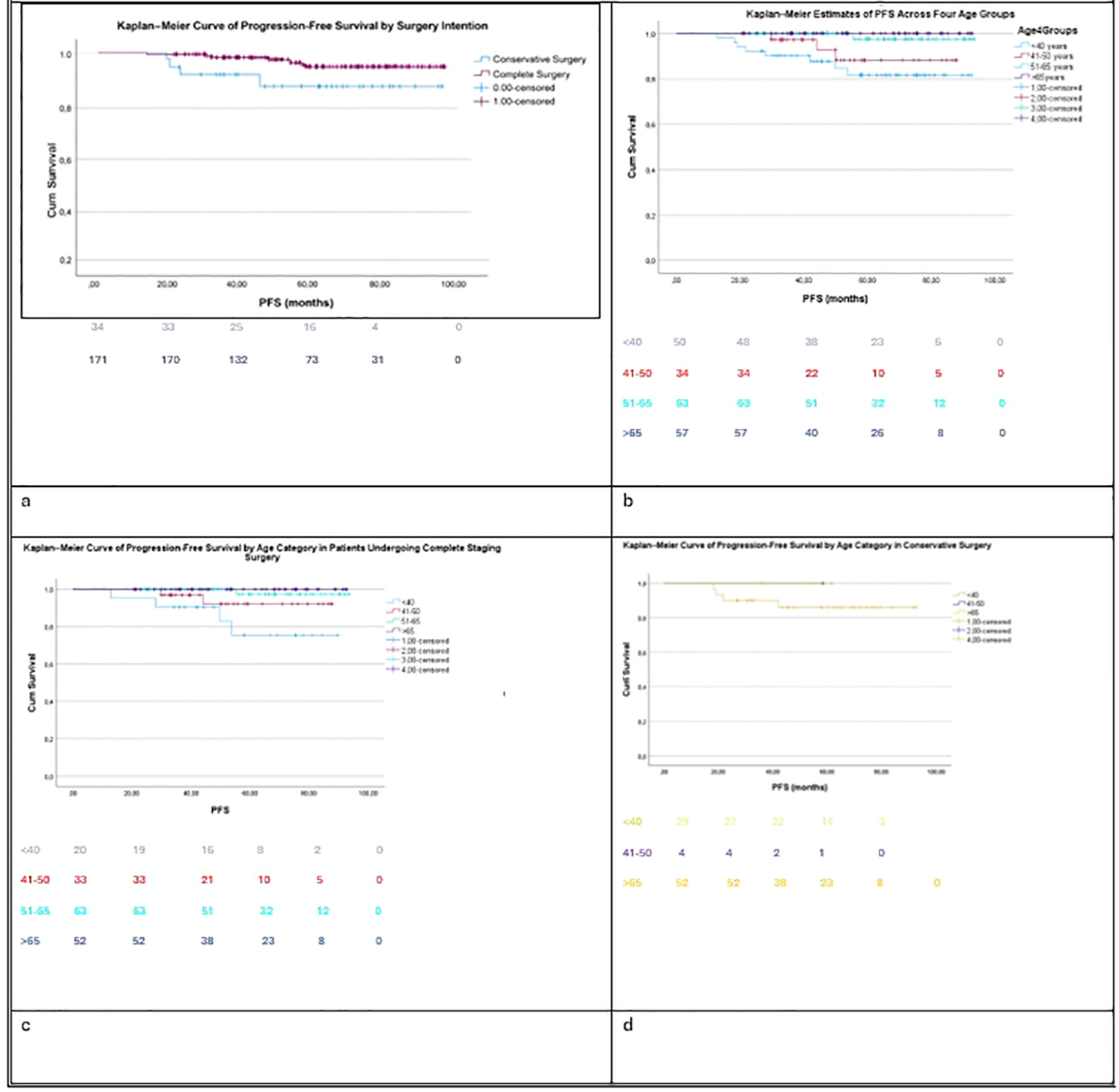
Kaplan-Meier curve for progression-free survival: **(A)** by surgical intention (p=0.08) **(B)** by age category (p=0.002) **(C)** in conservative surgery (p=0.001) **(D)** in complete surgery (p=0.721).

The Kaplan–Meier analysis showed a trend toward a difference between conservative and radical surgery (p=0.08). Although not statistically significant, the small sample size in the conservative group increases the risk of statistical error. PFS was slightly longer for patients who underwent complete staging surgery (median 90 months) compared with those who received conservative surgery (median 85 months (**Figure 1**). Furthermore, recurrence was not found to negatively impact overall survival (p=0.41).

### Survival by age and surgical approach

Among 208 patients, 51 were aged ≤40 years, 35 were aged 41–50 years, 64 were aged 51–65 years, and 58 were aged >65 years. Conservative surgery was performed in 36 patients (30 aged ≤40 years, 1 aged 41–50 years, and 5 aged >65 years), while 172 patients underwent complete staging surgery.

Recurrence occurred in 4 patients (1%) after conservative surgery in the ≤40-year group and in none of the older age groups (p = 0.417). Five-year survival was 100% for patients aged ≤40 years and 41–50 years but decreased to 80% in those aged >65 years (p = 0.014). At the end of follow-up, all patients aged ≤40 years and 41–50 years were alive, compared to 60% in the >65-year group (**Table 3**).

**Table 3:** Survival by age category.

Age	≤ 40 years	41–50 years	51–65 years	>65 years	
Total 208 (patients)	51 (24.5%)	35 (16.8%)	64 (30.8%)	58 (27.8%)	
Conservative surgery	30 (58.82%)	1 (1.56%)	0	5 (8.6%)	0.001
Relapse (yes)	4 (13.3%)			0	0.417
5 Years survival (yes)	17 (100%)			1(100%)	
Alive at the end of the study (yes)	30 (100%)	1 (100%)		3 (60%)	0.001
Complete Surgery	21 (41.18%)	34 (97.14%)	64 (100%)	53 (91.24%)	0.001
Relapse (yes)	4 (19%)	2 (5.9%)	1 (1.6%)	7 (4.1%)	0.001
5 Years survival	11/11(100%)	11/11 (100%)	30/32 (93.8%)	22/24 (91.7%)	0.5
Alive at the end of the study (yes)	21 (100%)	33(97.1%)	61(95.3%)	48 (90.5%)	0.328

In the complete surgery group, recurrence rates were 19% in patients aged ≤40 years, 5.9% in those aged 41–50 years, 1.6% in those aged 51–65 years, and 4.1% in those aged >65 years (p = 0.001). Five-year survival remained high across all age groups (94.4–100%) with no significant difference (p = 0.43, **Table 2**).

### Kaplan–Meier analysis

Kaplan–Meier analysis demonstrated several factors associated with progression-free and overall survival.

PFS showed a trend towards better outcomes in patients who underwent complete staging surgery compared with those treated with conservative surgery approaches, although this difference did not reach statistical significance (p = 0.08, **Figure 1A**).

When stratified by age category, a significant difference in PFS was observed (p = 0.002, **Figure 1B**). Overall, younger patients had the highest relapse risk. When analyzing the two surgical methods separately, younger patients exhibited a higher recurrence risk in both conservative and complete surgery, with the difference reaching statistical significance in the conservative surgery group (**Figure 1C**, p = 0.001). In contrast, no significant age-related difference in PFS was observed in the subgroup treated with conservative surgery (p = 0.72, **Figure 1D**).

**Figure 2:**
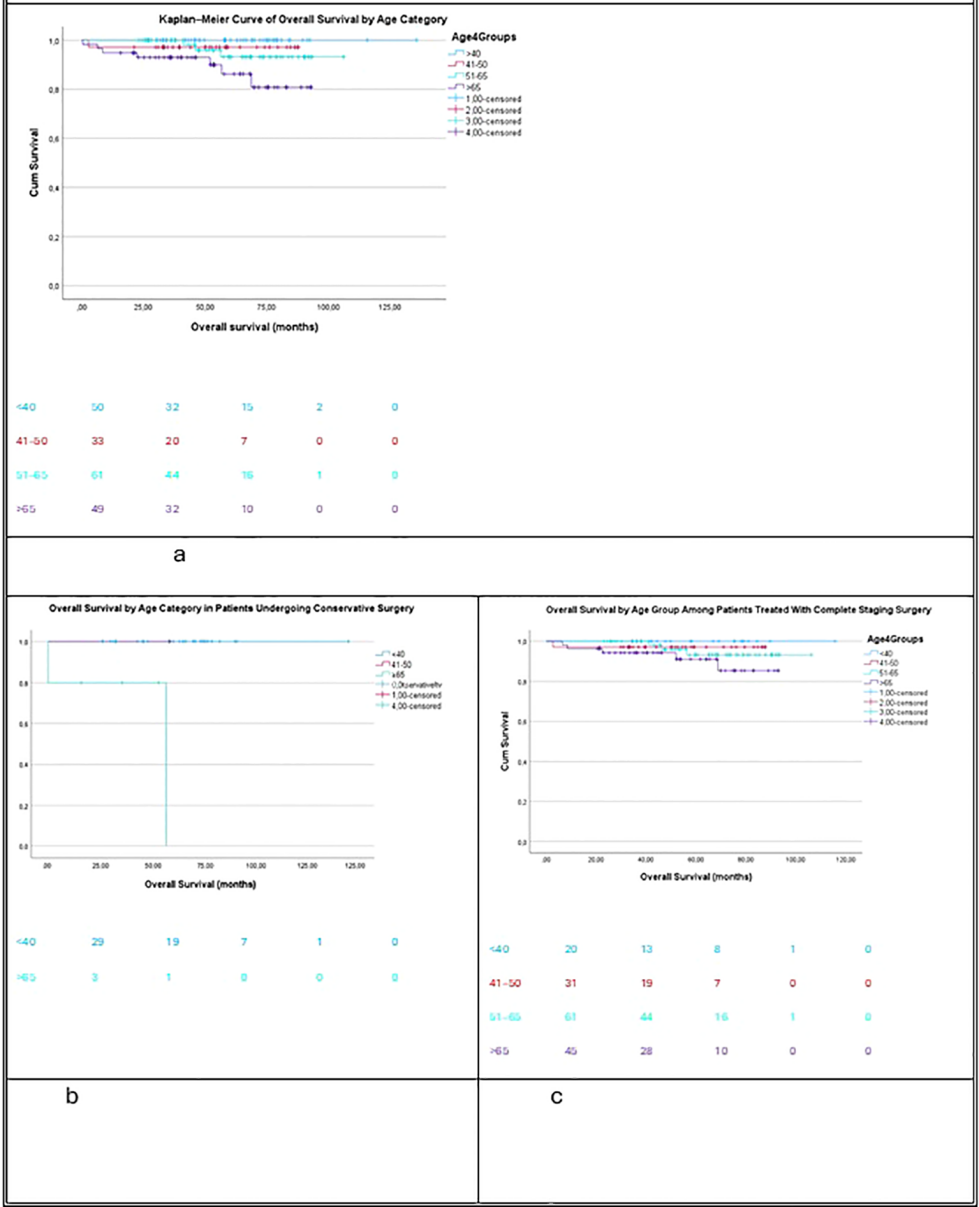
Kaplan-Meier overall survival: **(A)** by age category (p=0.02) **(B)** in conservative surgery (p=0.001) **(C)** in complete surgery (p=0.287).

Analysis of OS also revealed significant age-related variation. OS differed across age categories (<40, 41–50, 51–65, and >65 years), with elderly patients demonstrating more unfavorable outcomes (p = 0.02, **Figure 2A**). In the conservative surgery subgroup, younger age was strongly associated with improved OS (**Figure 2B**, p = 0.001). However, among patients who underwent complete staging surgery, OS did not significantly differ by age category (**Figure 2C**, p = 0.287).

### Cox regression analysis

In univariate cox-regression age at first surgery was a significant predictor for relapse (0.918 (95% CI: 0.877–0.962, p<0.001), indicating that for each additional year of age, the risk of relapse decreased by approximately 8%.

In the multivariable Cox proportional hazards model for PFS, higher age at first surgery was associated with a lower hazard of progression (HR per year = 0.893, 95% CI 0.842–0.947, p <.001). Conservative surgery (vs. complete surgery) was not associated with PFS (HR = 3.274, 95% CI 0.759–14.128; p = .112).

Age at first operation was significantly associated with survival (HR = 1.078, 95% CI: 1.028–1.130, p = 0.002), whereas recurrence, conservative surgery, and histology were not significant predictors. In the multivariate model, age remained the only independent predictor (HR = 1.068, 95% CI: 1.018–1.119, p = 0.007). Conservative surgery (HR = 0.820, p = 0.852) and recurrence (HR ≈ 0, p = 0.990) were not associated with survival, likely due to sparse events and wide confidence intervals.

To assess the safety of conservative surgery in patients more likely to have comorbidities, specifically those aged 65 years and older, we performed a Cox regression analysis for this age category.

The results showed that conservative surgery was not significantly associated with OS (HR = 0.562, 95% CI 0.112–2.822, p = .484) or PFS (HR = 0.450, 95% CI 0.132–1.537, p = .202) in this age category.

## Discussion

BOTs are characterized by excellent five- and ten-year survival rates, reaching up to 97% (5). In our study, overall survival was 94.1% high with no deaths observed among patients younger than 40 years and a slight decrease to 91.4% in patients older than 65 years. These findings highlight the generally favorable prognosis of BOTs across all age groups, although advanced age appears to indicate a modest increase in mortality risk. This may reflect the impact of comorbidities and competing causes of death rather than tumor biology itself. Importantly, the survival advantage in younger patients underscores the safety of conservative surgical approaches in this population, while careful selection and long-term follow-up remain essential for older patients.

The relapse risk was 11.4% after conservative surgery and 3.6% after complete surgery. These findings are consistent with the existing literature, which reports a recurrence risk of 10–20% following fertility-sparing surgery and approximately 5% after complete surgery (6).

The risk of malignant transformation is low, historically being described elsewhere as around 0.7% (8–10). Clinical surveys likely underestimate the true rates of recurrence and malignant transformation, primarily because of insufficient long-term follow-up. Longacre et al. describes a malignant transformation to low-grade serous carcinoma in 6.8% of cases at intervals of 7 to 288 months, associated with increased tempo of disease and decreased survival (10, 11). Recurrence can occur more than 5 years after diagnosis (32%), with 10% of recurrences observed beyond 10 years. These prolonged intervals raise the question of whether some tumors develop *de novo* rather than representing true relapses (12).

Numerous studies have investigated the role of surgical extent on overall survival. Although fertility-preserving surgery is consistently associated with a higher risk of recurrence, recurrence does not appear to negatively influence OS (5, 13).

A systematic review and meta-analysis reported a significantly lower risk of recurrence among women who underwent hysterectomy compared to those who preserved the uterus. However, this apparent advantage may be overestimated because ovarian preservation, rather than uterine preservation, is a well-established risk factor for BOT recurrence (14). A more recent meta-analysis attempted to clarify the safety of uterine preservation in patients with BOT, aiming to reduce surgical morbidity. It remains undetermined whether, in postmenopausal women with BOT, hysterectomy should be routinely recommended, particularly since hysterectomy does not appear to affect the risk of all-cause mortality or disease-specific mortality (6, 15).

In our study, we found no significant differences in recurrence risk or survival between conservative and complete surgery. Our results align with the findings of Raimondo D et al, that showed that uterine preservation did not increase the risk of recurrence or death from any cause compared with hysterectomy among postmenopausal women (16). In our cohort, the number of patients who underwent bilateral oophorectomy with uterine preservation was too small to draw definitive conclusions; however, we similarly observed no differences in relapse risk or survival between complete surgery and more conservative approaches.

Most patients in our study underwent complete surgery. Only 17.5% received conservative surgery, defined as uterus-preserving surgery with at least one ovary retained, and only five patients (2.4%) underwent unilateral salpingo-oophorectomy with uterine preservation. Even among younger patients, the rate of complete surgery was high: 41.2% of patients under 40 and 97.14% of those aged 41–50 underwent radical procedures. This pattern reflects Swedish clinical guidelines (17). These guidelines recommend that BOTs in patients without pregnancy desire be managed surgically according to principles applied in invasive epithelial ovarian cancer, except that lymphadenectomy is not performed. Furthermore, the evidence is insufficient to recommend systematic completion surgery after childbearing in recurrence-free patients who initially underwent conservative surgery (18, 19). Although recurrence risk is higher without completion surgery, most recurrences remain of borderline type. Therefore, current Swedish recommendations advise waiting until a recurrence occurs (1). Despite this, many patients are undergoing completion surgery after finishing childbearing, which may explain the high proportion of radical surgical procedures observed in patients aged 40–50.

Historically, clinical practice has focused more on fertility preservation than on endocrine preservation, despite the profound implications of surgically induced menopause occurring before the natural age of menopause (20). When analyzing postmenopausal patients, where fertility and endocrine function are no longer relevant, the need to limit the extent of surgery may be greater due to patient comorbidities. This raises an important clinical question from an oncological perspective: can a more limited surgical approach be safely offered in this population as well?

International guidelines vary substantially in their recommendations for hysterectomy among postmenopausal women with BOT. The National Comprehensive Cancer Network (NCCN) recommends hysterectomy, while the British Gynecological Cancer Society (BGCS) does not. The Collège National des Gynécologues et Obstétriciens Français (CNGOF) recommends hysterectomy only for the endometrioid histotype (13). The European Society for Medical Oncology–European Society of Gynecological Oncology (ESMO–ESGO) consensus does not provide specific recommendations for or against hysterectomy in postmenopausal women, instead allowing this decision to be made on an individualized basis (21–24). The considerable heterogeneity in indications for hysterectomy among postmenopausal women with BOT highlights the current lack of robust data on how hysterectomy alone influences survival outcomes.

In our study, we found that the risk of relapse was associated with age, with younger patients experiencing a higher risk of recurrence in both treatment strategies. Interestingly, this association reached statistical significance in the complete surgery group but not in the conservative surgery group. However, the conservative group included a small number of patients, and therefore, statistical interpretations should be made with caution. On the other hand, when we analyzed survival, the pattern was reversed, with reduced survival observed in older patients in both surgical strategies.

The finding that younger patients had a higher relapse risk, regardless of surgical approach, was unexpected for us as well. Unfortunately, our dataset does not include information on surgical approach (minimally invasive versus open surgery) or on surgical quality. In Sweden, ovarian cancer surgery is centralized, and patients with a preoperative suspicion of malignancy are referred to tertiary centers. However, borderline ovarian tumors are often difficult to diagnose preoperatively. This may influence where patients undergo surgery. In our cohort, 170 patients (64.6%) were operated at Lund University Hospital, while 93 patients (35.4%) underwent surgery at regional hospitals. It is therefore plausible that a larger proportion of younger patients were treated outside tertiary centers due to a lower preoperative suspicion of cancer in this age group. Such a pattern could have introduced variation in surgical expertise, which in turn may partly explain the higher relapse risk observed among younger patients.

In our study, age at first surgery emerged as significant and the only predictor of survival, while the extent of surgery did not appear to influence long-term outcomes. Conservative surgery was associated with a slightly higher recurrence rate but maintained excellent overall survival, supporting its use even in older patients to minimize surgical morbidity. Important limitations of our study include its retrospective design and the small number of recurrence events. Future research should focus not only on oncologic outcomes but also on quality-of-life measures, including the impact of endocrine function preservation, postoperative complication and morbidity secondary to extensive surgery.

A major strength of this study is the relatively large sample size and the fact that all patients were diagnosed and treated at a tertiary oncological center with access to a dedicated pathology team, ensuring high diagnostic accuracy and standardized management.

The main limitations of this study include its retrospective design and, for some patients, a relatively short follow-up period, which may affect the evaluation of long-term outcomes such as late recurrences. Another limitation is that we used ECOG performance status as a proxy for comorbidity. Although ECOG is routinely applied in clinical practice, it does not capture comorbidity burden as comprehensively as validated indices such as the Charlson Comorbidity Index. Because the study is retrospective, a more detailed comorbidity measure could not be applied, which limits our ability to fully adjust for this source of bias.

## Conclusion

Conservative surgery appears to be a safe alternative to complete staging surgery for borderline ovarian tumors across age groups. While age remains the strongest prognostic factor, the overall outcomes suggest that conservative management can be considered in appropriately selected patients. Careful patient selection and individualized clinical assessment are essential to ensure an optimal balance between oncologic safety and preservation of quality of life.

## Data Availability

The raw data supporting the conclusions of this article will be made available by the authors, without undue reservation.
